# Patient Age, Race and the Type of Diabetes Have an Impact on the Presenting Symptoms, Latency Before Diagnosis and Laboratory Abnormalities at Time of Diagnosis of Diabetes Mellitus in Children

**DOI:** 10.4274/jcrpe.v1i5.227

**Published:** 2009-08-03

**Authors:** Anıl R. Kumar, Paul B. Kaplowitz

**Affiliations:** 1 Department of Pediatrics, Virginia Commonwealth University, Medical College of Virginia, Richmond, VA, USA; 2 Department of Endocrinology, Children’s National Medical Center, Washington, DC, USA; +00 804−747−5087+00 804−828−3256arkumar@mail2.vcu.eduPO Box 980140, West hospital, 15th Floor, Richmond VA, USA 23298−0140

**Keywords:** type 1 diabetes, type 2 diabetes, new onset, hemoglobin A1c

## Abstract

**Objective**: To correlate the presence and duration of the symptoms with laboratory data in children with new onset diabetes mellitus (DM) and to determine the impact of gender, race, age, and type of diabetes on these relationships.

**Methods**: This was a single institution prospective study in which we asked the families of 112 children with new−onset DM a standard set of questions concerning the presence and duration of symptoms. We then reviewed selected laboratory data and explored the relationships between the symptoms, laboratory findings, gender, age, race, type of diabetes (T1DM or T2DM), and presence or absence of a history of diabetes in a close relative.

**Results**: Over 90% of patients had polyuria and polydipsia (mean duration 17 and 19 days), but only 50% of the families sought medical attention for this complaint. Children less than 5 years of age and African American children with T1DM were more dehydrated at presentation. More profound acidosis was seen in patients of younger age (<5 years), those with greater weight loss (9% or higher), and those with higher initial serum glucose (p<0.01). Mean hemoglobin A1c (HbA1c) was close to 11% for each subgroup and strongly correlated with the proportion of weight loss (p=0.0015), but not with the initial blood glucose, corrected serum sodium, or BUN levels.

**Conclusions**: Parents of children with new onset DM might not report polyuria or polydypsia as their main concern when they seek medical attention, so primary care physicians must be alert to the diagnosis of diabetes in any child with significant weight loss. Young children (<5 years old) and African American children with new onset T1DM are more dehydrated and young children (<5 years old) are more acidotic.

**Conflict of interest:**None declared.

## INTRODUCTION

Diabetes mellitus (DM) is one of the most common endocrine conditions and the incidence and prevalence of both type 1 DM (T1DM)  and type 2 DM (T2DM)  are increasing among children (1, 2). The initial complaint and presenting symptoms vary considerably from only mild symptoms to diabetic ketoacidosis (DKA) and shock. Some children are described by their parents to be symptomatic for only a few days prior to diagnosis, while others have had symptoms for several weeks. It is not clear to what extent a prolonged symptomatic period predicts the severity of dehydration or laboratory abnormalities at diagnosis. Furthermore, only a few previous studies have reported laboratory data and presenting symptoms in new−onset childhood diabetes. These were over a decade ago and did not examine the impact of race on the presentation of T1DM, nor did they include T2DM as a separate group. It is possible that increased provider and public awareness of diabetes as well as the increased incidence of T2DM among children might have had an impact on the initial presentation of diabetes in children. To address these issues, we collected data on initial symptoms, symptom duration, and initial laboratory data on all newly diagnosed patients with diabetes at our institution over a period of 18 consecutive months and explored relationships between these variables, and the possible impact of age, race and gender, presence of diabetes in a close relative, and the type of diabetes (T1DM or T2DM) on the type and duration of symptoms as well as extent of laboratory abnormalities. 

## METHODS

This study was approved by the Institutional Review Board of the Virginia Commonwealth University Health System. 

Between March 2002 and July 2003, 112 families of children with new onset DM seen at the Virginia Commonwealth University Health System were asked a standard set of questions (in most cases within 24 hours of diagnosis). The demographics of the study sample match the population served by the hospital and the entire community it serves. All the 112 children who were eligible during this time period were enrolled in the study. They were asked  if any of the symptom(s) that brought them to medical attention included polyuria, polydipsia, nocturia, polyphagia, abdominal pain, unusual fatigue, vomiting, and genital discharge or rash, all of which are known to be associated with DM. They were then asked the duration of each symptom. The presence of weight loss was estimated as the difference between admission weight and parental recall of the child’s weight at a recent (1−4 months preceding) health care visit. Family history for T1DM and T2DM (adult onset was assumed to be T2DM) was recorded. For analysis, we accepted only those with a history of T1DM or T2DM in a first or second degree relative (siblings, parents, grandparents, aunts, and uncles) as being a positive family history in a close relative.

A review of the initial laboratory tests included blood glucose, electrolytes, BUN, hemoglobin A1c (HbA1c), and urine ketones. Most of the HbA1c tests were done at the MCV Hospital using the HPLC assay (Primus) that has a normal range of 4.3−5.5%. The corrected sodium (Na) was calculated for each patient using the formula (Na + (blood glucose−100/1.6)).  Elevated corrected Na, like elevated BUN, reflected a greater degree of dehydration. We did not record pH values since they were not available for many patients who presented without DKA, but used the serum bicarbonate (HCO3) level as an index of the severity of acidosis. 

We defined a subset of patients (all of whom were African American) as having T2DM if they were obese and had acanthosis nigricans. Since most but not all patients with T2DM have acanthosis nigricans (3), it is possible that a few of the African American children classified as T1DM may have actually had T2DM.  Clinical signs helpful in distinguishing T2DM from T1DM are obesity and signs of insulin resistance (acanthosis nigricans, hypertension, polycystic ovary syndrome). Patients with T2DM frequently have elevated C−peptide levels. The absence of auto−antibodies to insulin, to islet cells, or to glutamic acid decarboxylase is also typical in most (but not all) cases of diabetes that are classified as T2DM (11). We did not measure antibody, C−peptide or insulin level in our patients. All these patients continue to show signs of insulin resistance on follow up for 2−3 years.

**Statistical Methods**

Mean values were calculated for the duration of each symptom and laboratory parameter. The entire group was then stratified according to race (African American or Caucasian), type of diabetes (T1DM or T2DM), age group (≤5 years, 6−12 years and ≥13 years), and family history of diabetes (present or absent). The mean values were compared by ANOVA. Linear regression analysis was applied to individual variables using Pearson correlation coefficients. The p values were defined as statistically significant when <0.05.  Nonsignificant values were denoted as NS.

## RESULTS

Of the 112 children in the study, 20.5% were younger than 5 years of age, 47.3% were between 6 and 12 years, and 32.1% were older than 13 years of age. Both genders were similarly represented (47% males and 53% females). T1DM was diagnosed in 56% of Caucasian children and 24% of African American children. T2DM was diagnosed in 20% of children, all of whom were African American.  

Polydipsia, polyuria and nocturia were the most common symptoms and were reported in 89−93% of patients while polyphagia was noted in only 19%. The mean duration of these symptoms was 16.8 days for polyuria, 17.3 days for nocturia and 19.3 days for polydypsia (Table 1). Fatigue (63%) and weight loss (68%) were the next most common symptoms, while abdominal pain (38%) and vomiting (45%) were reported less frequently. Among the patients with vomiting, there was a single episode of emesis in 19 (38%), often more than a day before they sought medical attention. In 31 patients (62%), there were multiple episodes of emesis.  Genital rash or discharge was noted in 10% of all patients, and in 19% of females.    

Only 50% the parents reported taking their child to a physician because of polyuria and polydypsia and surprisingly, only 8% took their child for medical attention due to weight loss (Table 2).  Nineteen percent reported their major concern was their child’s color and/or appearance.

Table 3 shows duration of polydipsia, estimated % weight loss, and laboratory values when the entire group was stratified by race, type of diabetes, age group, and the presence or absence of diabetes in a first or second−degree relative.  Mean HbA1c was 11.3% for the entire group and showed similar values (10.75−11.8%) in all subgroups. Mean blood glucose at diagnosis was 584 mg/dl. A trend was apparent for African American children with T1DM to present with greater dehydration (higher corrected Na and BUN) and acidosis (lower HCO_3_) than either Caucasian children with T1DM or all children with T2DM. Caucasian children with T1DM had the shortest duration of polydypsia (14.2 days), followed by African American children with T1DM (19.5 days) and children with T2DM (25.5 days). Gender had no impact on presenting symptoms, duration of symptoms or laboratory parameters except that males had a somewhat lower HCO_3_ at diagnosis (15.9) than females (18.3). Family history of diabetes had little impact on duration of polydipsia, although weight loss and laboratory abnormalities were slightly more severe in patients without a family history of diabetes. When stratified by age, younger children (<5 years of age) had greater weight loss (10.6%), were more acidotic (HCO_3_=15.3) and had slightly higher corrected serum Na (145 mmol/L) than did older children (8.1% body weight loss, HCO_3_=20.3 mmol/L, and corrected Na=141.4 mmol/L) (p<0.01), but mean HbA1c in the younger children (10.75%) was the lowest of any age group (11.4% in 6−12 year olds, and 11.5% in ≥13 year olds). Of the total 66 Caucasians with T1DM, 25 children presented with DKA and of the total 25 black children with T1DM, 11 children presented with DKA.

To look for relationships between the different variables presented in Table 3, linear regression analysis was performed among all combinations of variables using Pearson correlation coefficients (r). The relationship between the variables analyzed was complex, but certain significant correlations (p<0.05) were noted.

1) Younger age (p=0.007, r=0.26), greater weight loss (p<0.001, r=0.54), and higher serum glucose (p<0.001, r=−0.39),  but not duration of polydipsia (NS, r=0.12) correlated with a greater degree of acidosis (lower serum HCO_3_).

2) Higher glucose (p<0.001, r=0.67), lower HCO_3_ (p<0.001, r=−0.42), and higher corrected serum Na (p<0.001, r=0.37) correlated with a higher BUN.

3) HbA1c strongly correlated with % weight loss (p=0.0015, r=0.35) and to a lesser 

degree with duration of polyuria (p=0.03, r=0.21) and polydipsia (p=0.017, r=0.24), but not with serum glucose (NS), corrected serum Na (NS), or BUN (NS).

4) Duration of polydipsia correlated with weight loss (p=0.0064, r=0.31) and HbA1c (p=0.017, r=0.24), and duration of polyuria correlated with weight loss (p<0.04, r=0.3) and HbA1c (p=0.03, r=0.21).

There was no correlation between the degree of ketonuria (mild, moderate, severe) and duration of polyuria and polydipsia.

**Table 1:**
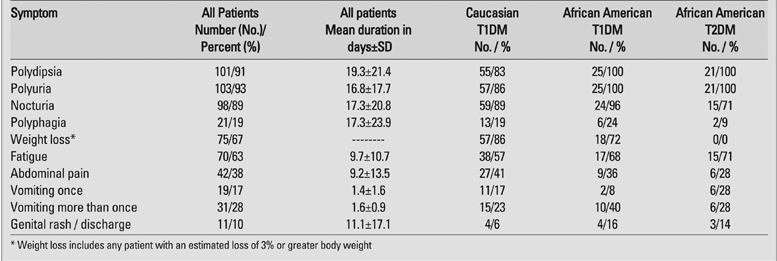
Frequency of the presenting symptoms

**Table 2:**
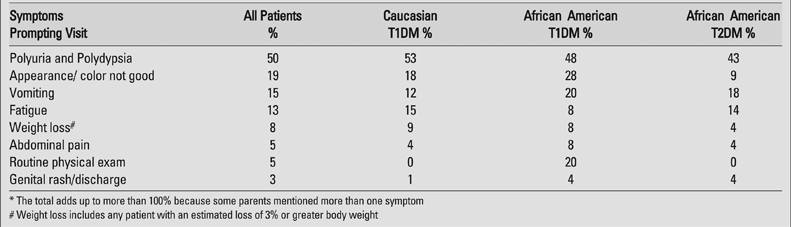
Symptoms reported as prompting parents to seek medical attention*

**Table 3:**
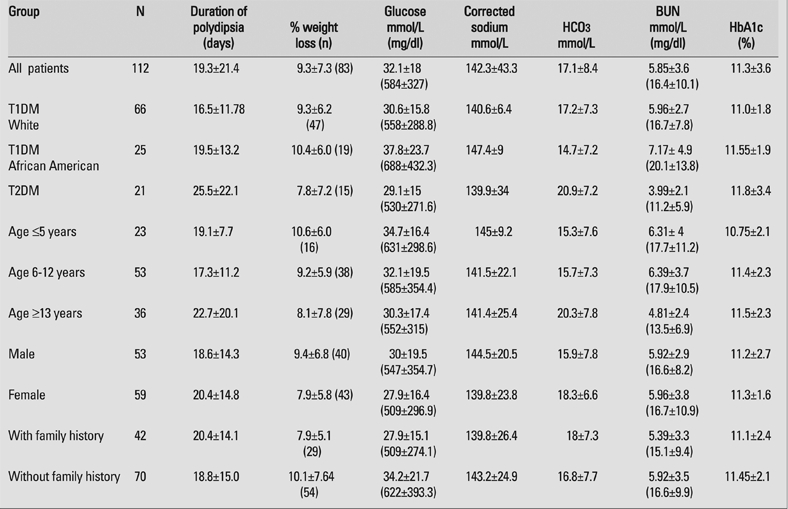
Duration of polydipsia, % weight loss, and selected laboratory tests for different patient subgroups (mean ± SD)

## DISCUSSION

Only a few previous studies have examined laboratory data and presenting symptoms in new−onset childhood diabetes. These were over a decade ago and did not examine the impact of race on the presentation of T1DM. These reports  also did not include T2DM as a separate group. It is possible that increased provider and public awareness of diabetes as well as the increased incidence of T2DM among children might have had an impact on the initial presentation of diabetes in children. Contrary to this hypothesis, we found that the prevalences of polyuria (91%) and polydipsia (93%) in our population were comparable to that found about a decade previously by Levy−Marchal (90% had polyuria) (1) and Drash (90% in males and 88% in females) (4). We also found a similar proportion of children with weight loss (68%) as did Levy−Marchal (63%) but this was less common than in the data from Drash (80% in males and 84% in females). The mean percent weight loss in our study (9.3%) was almost identical to that reported by Levy−Marshall (9.4%).  However, they reported a smaller percent weight loss (7.9%) in the youngest children (≤4 years of age) while we found the highest percent weight loss (10.6%) in children ≤5 years of age. This discrepancy could be due to the fact that we relied on parental reports of previous body weight (within 4 months of presentation).                               

Nearly half of parents in our study reported vomiting in the few days prior to presentation and this finding was often attributed to a viral illness. However 38% of children vomited only once, and in many cases this was more than a day prior to admission. Nevertheless, emesis was a late feature of diabetes in almost all cases. 

The mean durations of polyuria and polydypsia prior to the diagnosis of diabetes were variable but typically in the 2−3 weeks range. Symptom duration is only an approximation, as many parents did not recall precisely when a particular symptom came to their attention. However our data are remarkably similar to that of a recent study of 109 children in Spain (7). They found that 95.8% of all children had polyuria and polydypsia. The mean duration of these symptoms was 25.4 days in children between 10−14 years of age; 21.7 days in children aged 5−9 years and 13.6 days in those <4 years old (7). In contrast, we found that the mean duration of polydypsia was 19.1 days in our youngest age group (≤5 years). Mean duration for older children (17.3 days in 6−12 year olds and 22.7 days in children ≥13 years of age) was similar to the Spanish data. In a large study of over 2000 children with T1DM, Neu et al (3) reported that the average duration of symptoms (11.2 days) was shorter among young children (<4 years of age) than older (15.2 days). However, a greater proportion of young children (36%) had DKA, while a smaller proportion (23%) of older children presented with DKA. Their data suggest a more robust tempo of disease progression among young children. Similarly, Roche et al (8) reported that children <2 years of age are more likely to present with moderate to severe DKA. Our data offer a mixture of insights into these questions. In our study, the duration of polydypsia was the same for the young as for older children, but HbA1c was actually lower in the young children. In contrast, mean HCO_3_ was lowest in our young patients (<5 years of age), percentage of body weight loss was also greatest in this age group, and their mean serum glucose was highest compared to 6−12 year olds and older (>13 years) children. Similar to our data, Levy−Marchal et al found a higher proportion of children with HCO_3_  less than 18 mmol/L in the 0−4 year age group (66%), as compared to the 10−19 year olds (30−48%) (1). Whether or not these features really differ remains unclear. Despite this uncertainty, it is clear that the presenting symptoms and duration of symptoms have changed little if any over the previous decade despite an increase in the incidence of T2DM.

When we stratified our data by race and type of diabetes, we found that African American children with T1DM had a longer duration of polydipsia, that they were sicker and more dehydrated than Caucasian children. There are many possible explanations for this including limited access to health care among minority patients (6, 7, 8, 9). Our data are consistent with the hypothesis that it takes longer for the parents of African American children to seek medical attention. On average, African American children had 5 more days of polydypsia than did Caucasian children. Interestingly, mean HbA1c was only 0.5% higher in African American children with T1DM than in Caucasian children with T1DM. It is possible that some or all of this delay could arise from inadequate health insurance. We did not collect this data from our subjects but in a previous study, Mallare et al (9) found that 38% of 139 new onset T1DM children (0.5−18 years of age) presented with DKA. Those with Medicaid or no insurance were more likely to present with DKA (62%) than were patients with private insurance (34%). They concluded that lack of private insurance (a proxy for socioeconomic status) and young age were risk factors for DKA. 

We also found that children with T2DM, all of whom were African American, had a longer duration of symptoms, little or no acidosis, and less dehydration when compared to all children with T1DM. This suggests that children with T2DM experienced a slower metabolic deterioration than children with T1DM. Despite this fact, 20% of children with T2DM in our study presented with DKA. This frequency is slightly higher than figures reported in previous studies (5−10%) (5). We do not have an explanation for the increased prevalence of DKA, but it could be due to the longer duration of symptoms or more serious islet destruction.  

One of the more intriguing findings of our study is that mean HbA1c was close to 11% for the entire group and for every subgroup.  Although Levy−Marchal et al (1) did not report HbA1c values; they reported mean glycosylated hemoglobin of 212% of the mean reference value which is similar to a HbA1c value of 11%. In a series of patients from Pittsburgh, mean HbA1 at diagnosis was 13.4% in males and 14.9% in females. Using the formula HbA1c=(HbA1−0.19)/1.2, this approximates to an HbA1c of 9.5% in males, and 10.8% in females.  In our study, we did not find a male−female difference in mean HbA1c at the time of diagnosis. Barker et al (6) reported an average HbA1c in symptomatic children with T1DM of 10.9%. This was greater than that of children with pre−diabetes (HbA1c:7.2%), patients who eventually progressed to overt diabetes. The similarity in all these studies and in our different subgroups suggests that regardless of age, gender, race and type of diabetes, all children have experienced a similar “glycemic exposure” at the time of diagnosis.  

In contrast to our expectation, a family history of diabetes did not appear to result in earlier diagnosis. Patients with a positive family history of diabetes had a slightly longer duration of polydypsia than did patients without this history. A family history of diabetes may have mitigated against the severity of disease, since proportion of  weight loss and laboratory abnormalities were less severe in the patients with a positive family history of diabetes. 

By linear regression analysis we found that children who had a longer duration of polydypsia had a greater percentage of weight loss but not higher glucose, BUN, corrected Na, or lower HCO_3_ levels.  Thus a longer duration of symptoms did not necessarily equate with severity of illness. This suggests that the progression of disease may have been slower in children with a longer symptomatic period. The children who had more weight loss were significantly more acidotic and had higher HbA1c levels, but were not more dehydrated based on the corrected Na and BUN. Their weight loss may have been due to loss of body fat rather than acute dehydration. 

Of major importance for primary care physicians, younger patients (≤5 years old) were more acidotic and more dehydrated than older patients. A prolonged period of polyuria and polydipsia (>3 weeks) and more than 9% weight loss in children indicated a greater likelihood of DKA and a need for intensive care admission.  

To our knowledge this is the first study in which parents of children with newly diagnosed diabetes were asked what prompted them to seek medical attention. Although about 90% of the patients had polyuria and polydypsia, only 50% of parents sought medical care because of it. Therefore, primary care providers should have a high index of suspicion for diabetes in any child presenting with symptoms such as fatigue, poor appearance, and particularly weight loss. Ideally, blood glucose and/or urine testing should be done immediately. Unfortunately, several of the patients with the classic symptoms of DM are sent to a commercial lab for blood tests, and by the time the results are received a day or two later, the child will have progressed to DKA. This is especially common among young patients (9). The overall mortality rate from pediatric DKA in the United States is approximately 0.15%, and cerebral edema accounts for 57−87% of deaths related to DKA (10). Earlier diagnosis and referral can prevent DKA and its complications.

In summary, the most common presenting signs and symptoms of DM in children (polyuria, polydypsia and nocturia) have changed little over the past decade despite a dramatic increase in the incidence of T2DM. However,parents rarely bring their child to the physician for these symptoms. Rather they complain of fatigue, weight loss, a change in appearance, or vomiting (even if infrequent). Primary care physicians should be very astute to these complaints especially in the young child and those with suspected weight loss. The diagnosis of diabetes can be enhanced by rapid glucose testing in the office followed by referral and confirmation by laboratory studies at the time of hospitalization. Preventive health counseling should include discussions of the risk factors for T2DM (obesity, acanthosis nigricans, membership in high−risk minority populations, and family history of T2DM), and the signs and symptoms of diabetes in children.
